# Supplementing organic-complexed or inorganic Co, Cu, Mn, and Zn to beef cows during gestation: postweaning responses of offspring reared as replacement heifers or feeder cattle

**DOI:** 10.1093/jas/skab082

**Published:** 2021-03-14

**Authors:** Kelsey M Harvey, Reinaldo F Cooke, Eduardo A Colombo, Bruna Rett, Osvaldo A de Sousa, Lorin M Harvey, Jason R Russell, Ky G Pohler, Alice P Brandão

**Affiliations:** 1 Department of Animal Science, Texas A&M University, College Station, TX 77845, USA; 2 Prairie Research Unit, Mississippi State University, Prairie, MS 39756, USA; 3 Faculdade de Medicina Veterinária e Zootecnia, Universidade Estadual Paulista, Botucatu, SP 18618-970, Brazil; 4 Pontotoc Ridge-Flatwoods Branch Experiment Station,Mississippi State University, Pontotoc, MS 38863, USA; 5 Zinpro Corporation, Eden Prairie, MN 55344, USA

**Keywords:** beef cows, gestation, offspring, physiology, production, trace minerals

## Abstract

One hundred and ninety nonlactating, pregnant beef cows (¾ *Bos taurus* and ¼ *Bos indicus*; 138 multiparous and 52 primiparous) were assigned to this experiment at 117 ± 2.2 d of gestation (day 0). Cows were ranked by parity, pregnancy type (artificial insemination = 102, natural service = 88), body weight (**BW**) and body condition score, and assigned to receive a supplement containing: (1) sulfate sources of Cu, Co, Mn, and Zn (**INR**; *n* = 95) or (2) an organic complexed source of Cu, Mn, Co, and Zn (**AAC**; Availa4; Zinpro Corporation, Eden Prairie, MN; *n* = 95). The INR and AAC provided the same daily amount of Cu, Co, Mn, and Zn, based on 7 g of the AAC source. From day 0 to calving, cows were maintained in a single pasture and segregated 3 times weekly into 1 of 24 individual feeding pens to receive treatments. Calves were weaned on day 367 (200 ± 2 d of age), managed as a single group for a 45-d preconditioning period (days 367 to 412), and transferred to a single oat (*Avena sativa* L.) pasture on day 412. Heifer calves were moved to an adjacent oat pasture on day 437, where they remained until day 620. Heifer puberty status was verified weekly (days 437 to 619) based on plasma progesterone concentrations. Steer calves were shipped to a commercial feedlot on day 493, where they were managed as a single group until slaughter (day 724). Plasma cortisol concentration was greater (*P* = 0.05) in AAC calves at weaning but tended to be less (*P* = 0.10) on day 370 compared with INR calves. Mean plasma haptoglobin concentration was greater (*P* = 0.03) in INR vs. AAC calves during preconditioning, and no treatment effects were noted (*P* = 0.76) for preconditioning average daily gain (**ADG**). Puberty attainment was hastened in AAC heifers during the experiment (treatment × day; *P* < 0.01), despite similar (*P* = 0.39) ADG between treatments from days 412 to 620. Expression of *myogenin* mRNA in the *longissimus* muscle was greater (*P* = 0.05) in INR vs. AAC heifers on day 584. No treatment effects were detected (*P* ≥ 0.24) for steer ADG from day 412 until slaughter, nor for carcass quality traits. Hepatic mRNA expression of *metallothionein 1A* was greater (*P* = 0.02) in INR vs. AAC steers on day 586. In summary, supplementing Co, Cu, Zn, and Mn as organic complexed instead of sulfate sources to beef cows during the last 5 mo of gestation did not improve performance and physiological responses of the steer progeny until slaughter, but hastened puberty attainment in the female progeny reared as replacement heifers.

## Introduction

The fetus relies on the dam for supply for trace minerals essential for its developmental processes, such as protein synthesis, bone formation, and lipid metabolism (Hidiroglou and Knipfel, 1981; Hostetler et al., 2003). Limited information, however, is available on how trace mineral nutrition of gestating cows impacts offspring productivity. Our research group recently investigated this subject by supplementing late-gestating beef cows with organic complexed sources of Co, Cu, Mn, and Zn (Marques et al., 2016), which are expected to have greater bioavailability compared to sulfates sources (Spears, 1996). Marques et al. (2016) reported that calves born from cows supplemented with organic complexed Co, Cu, Mn, and Zn were heavier at weaning and slaughter compared with calves from nonsupplemented cows, and had reduced incidence of bovine respiratory disease (**BRD**) compared with calves from both nonsupplemented and inorganic-supplemented cows. Authors attributed these outcomes to enhanced passage of Zn and Cu from maternal to fetal tissues, resulting in life-long programming effects on offspring productivity and health (Funston et al., 2010).

The fetus is sensitive to the effects of maternal nutrition from all stages between oocyte maturation through parturition (Wu et al., 2006), and Marques et al. (2016) evaluated trace mineral supplementation during the last 90 of gestation. Moreover, male and female offspring were reared for slaughter by Marques et al. (2016), while maternal nutrition and weaning BW have profound impacts on female reproductive development (Martin et al., 1992; Cushman and Perry, 2019). Therefore, we hypothesized that supplementing organic complexed Co, Cu, Mn, and Zn to beef cows during a greater period of gestation will improve offspring development beyond the findings from Marques et al. (2016), including hastened puberty attainment of heifer calves. To test this hypothesis, this experiment evaluated the effects of supplementing organic complexed or sulfate sources of Co, Cu, Mn, and Zn to beef cows during the second and third trimesters of gestation on postweaning responses of the female offspring reared as replacement heifers, and the male offspring reared as feeder cattle for slaughter.

## Materials and Methods

This experiment was conducted at the Texas A&M, Beef Cattle Systems (College Station, TX). All animals were cared for in accordance with acceptable practices and experimental protocols reviewed and approved by the Texas A&M, Institute of Animal Care of Use Committee (#2018/0093). This manuscript describes the postweaning responses of the female and male offspring reared, respectively, as replacement heifers and feeder steers for slaughter. A companion manuscript (Harvey et al., 2021) describes pre- and postpartum responses of cows, as well as offspring responses from birth through weaning.

### Cow management and dietary treatments

A full description of dietary treatments and management scheme applied to beef cows are described in the companion manuscript (Harvey et al., 2021). Briefly, 190 nonlactating, pregnant beef cows (average ¾ *Bos taurus* and ¼ *Bos indicus*; 138 multiparous, 52 primiparous) were assigned to this experiment at 117 ± 2.2 d of gestation (day 0 of the experiment). Cows were ranked by parity, pregnancy type (artificial insemination = 102, natural service = 88), body weight (**BW**) and body condition score, and assigned to receive a supplement containing: (1) sulfate sources of Cu, Co, Mn, and Zn (**INR**; custom blend manufactured by Anipro Xtraformance Feeds, Pratt, KS; *n* = 95) or (2) organic complexed source of Cu, Co, Mn, and Zn (**AAC**; Availa4; Zinpro Corporation, Eden Prairie, MN; *n* = 95). The INR and AAC provided the same daily amount of Cu, Co, Mn, and Zn, based on 7 g of the AAC source as in Marques et al. (2016). From day 0 to calving, cows were maintained in a single pasture and were segregated 3 times weekly into 1 of 24 individual feeding pens to receive treatments. Cows individually received supplement treatments (601 and 607 g of INR and AAC per cow each feeding; as-fed basis) and returned to pasture after their treatment was completely consumed.

After calving, cow-calf pairs were maintained in a single pasture and assigned to the general management of the research herd, which included free-choice inorganic trace mineral supplementation (Producers Special Pasture Mineral; Producers Cooperative Association, College Station, TX; containing 14% Ca, 7% P, 13% NaCl, 5% Mg, 9,900 mg/kg Zn, 2,500 mg/kg Cu, 100 mg/kg I, 4,000 mg/kg Mn, 26 mg/kg Se, 91 IU/g of vitamin A, 10 IU/g of vitamin D3, and 0.05 IU/g of vitamin E). This trace mineral supplement was the same fed to cows prior to the beginning of this experiment. Male calves were castrated using an elastic bander at ~30 d of age. All calves received vaccination against respiratory viruses (Triangle 5; Boehringer Ingelheim Animal Health USA Inc., Duluth, GA) and Clostridium (Covexin 8; Merck Animal Health, Omaha, NE) on day 345 of the experiment. Calves were weaned on day 367, when they were revaccinated against respiratory viruses (Titanium 5; Elanco Animal Health, Greenfield, IN) and Clostridium (Covexin 8; Merck Animal Health), and received an anthelmintic (Dectomax; Zoetis, Florham Park, NJ).

### Postweaning calf management

#### All calves

 After weaning, calves were transferred to a single 5.3 ha paddock for a 45-d preconditioning period (days 367 to 412). Calves had ad libitum access to mixed bermuda-ryegrass hay, water, and a total mixed ration (**TMR;**Table 1) containing the mineral and vitamin mix previously described (Producers Special Pasture Mineral; Producers Cooperative Association). On day 412, all calves were transferred to a single 52.6 ha oat (*Avena sativa* L.) pasture for a 25-d backgrounding phase as a single group, receiving the mineral and vitamin mix described (Producers Special Pasture Mineral; Producers Cooperative Association) and water for ad libitum consumption.

**Table 1.** Nutritional profile (dry matter basis) of feedstuffs offered to heifers and steers after weaning^1,2^

Item	Bermuda-Rye Grass Hay	TMR	Pasture A	Pasture B
Net energy for maintenance, Mcal/kg	1.00	1.76	1.36	1.22
Net energy for gain, Mcal/kg	0.44	1.13	0.78	0.62
Crude protein, %	8.70	16.4	22.3	13.6
Neutral detergent fiber, %	72.4	30.0	41.0	54.2
Ca, %	0.61	0.80	0.37	0.48
P, %	0.17	0.61	0.42	0.26
Mg, %	0.13	0.30	0.18	0.11
K, %	1.87	1.64	4.41	1.85
Na, %	0.06	0.23	0.23	0.06
Co, mg/kg	0.36	0.73	0.36	0.27
Cu, mg/kg	8.0	51	10	8
Fe, mg/kg	221	305	784	272
Mn, mg/kg	72	127	68	37
Se, mg/kg	0.07	0.68	0.25	0.18
Zn, mg/kg	30	165	30	36

^1^Values obtained from a commercial laboratory wet chemistry analysis (Dairy One Forage Laboratory, Ithaca, NY). Total Digestible nutrients were calculated according to equations described by Weiss et al. (1992). Net energy for maintenance and gain were calculated with equations described by the NRC (2000).

^2^Calves were weaned and had ad libitum access to mixed bermuda-ryegrass hay and a TMR during preconditioning (days 367 to 412). The TMR consisted of (as-fed basis) 31.8% cracked corn, 30.0% dried distillers grains, 28.8% alfalfa hay, 7.0% liquid molasses, and 2.1% of an inorganic mineral mix containing 14% Ca, 7% P, 13% NaCl, 0.27% K, 0.4% Mg, 0.25% Cu, 0.003% Se, 0.99% Zn, 90.91 IU/kg of vitamin A, 9.09 IU/kg of vitamin D3, and 0.045 IU/kg of vitamin E. On day 412, calves were transferred to a single oat pasture (*Avena sativa L*.; Pasture A), where steers were maintained until transport to the feedyard on day 493. Heifers were transferred to an adjacent oat pasture (Pasture B) on day 437 and remained there until day 620.

#### Heifer calves

On day 437, heifer calves (33 from INR cows and 45 from AAC cows) were moved to an adjacent 44.5 ha oat pasture where they remained until day 620. Heifers had ad libitum access to water and the mineral and vitamin mix previously described (Producers Special Pasture Mineral; Producers Cooperative Association) for ad libitum consumption.

#### Steer calves

On day 437, steer calves (50 from INR cows and 44 from AAC cows) remained in the same 52.6 ha oat pasture for an additional 56-d backgrounding period, with ad libitum access to water and the mineral and vitamin mix previously described (Producers Special Pasture Mineral; Producers Cooperative Association). On d 493, steers were loaded into a commercial livestock trailer (Legend 50’ cattle liner; Barrett LLC., Purcell, OK) and transported 202 km to a commercial feedyard (Graham Land and Cattle Company; Gonzales, TX). Upon arrival, steers were revaccinated against respiratory viruses (Bovi-Shield Gold 5; Zoetis) and Clostridium (UltraChoice 8; Zoetis), received an anthelmintic (Dectomax; Zoetis) and steroidal implant (Synovex S; Zoetis). Steers received another steroidal implant (Synovex Choice; Zoetis) on day 586. Steers were managed as a single group and received diets described in Table 2 until slaughter (day 724) at a commercial facility (STX Beef; Corpus Christi, TX).

**Table 2.** Ingredient composition (as-fed basis) of diets offered to steers in the feedlot^1^

Ingredients, % as-fed basis	A	B	C	D
Brewers grain	35.0	28.0	21.0	21.0
Cottonseed hulls	16.0	9.5	2.5	2.5
Dried corn	33.5	47.0	64.0	69.0
Rice bran	7.0	10.0	10.0	5.0
Liquid molasses	6.0	3.0	0.0	0.0
Mineral and vitamin mix^2^	2.5	2.5	2.5	2.5

^1^Steers were transported to the feedlot on day 493 where they remained until slaughter. Diet A, offered for 15 d on receiving; B, offered for 20 d after diet A; C, offered for 32 d after diet B; D, offered until slaughter.

^2^All diets included a customized blend of minerals, vitamins, and feed additives (Purina Animal Nutrition, Arden Hills, MN), which contained one-third of Zn, Mn, and Cu as metal:AA complex ratio (Zinpro Corporation, Eden Prairie, MN) and two-thirds as sulfate sources.

### Sampling

#### Feedstuffs

Samples of all ingredients fed to calves during the preconditioning and backgrounding were collected monthly, pooled across months, and analyzed for nutrient content by a commercial laboratory (Dairy One Forage Laboratory, Ithaca, NY). Nutritional profile of all ingredients is described in Table 1.

#### All calves during preconditioning and backgrounding

Calf BW was recorded on days 367 and 368 (averaged for weaning BW) and on days 411 and 412 (averaged as final preconditioning BW), which were used to calculate preconditioning average daily gain (**ADG**). Blood samples were collected on d 345, 367, 368, 370, 373, 377, 382, and 397 from 30 calves randomly selected from each treatment. Calves were observed daily for BRD signs from days 367 to 437 according to the subjective criteria described by Berry et al. (2004).

#### Heifer calves

Heifer BW was recorded and blood samples collected weekly from days 437 to 619. Heifer BW was also recorded on day 620 and averaged with day 619 values as heifer final BW. Heifer ADG was calculated using preconditioning BW and final BW. Growth rate of each heifer was also modeled by linear regression of BW against sampling days (days 437 to 619), and each regression coefficient was used as individual response. On day 584, 15 heifers from each treatment were randomly selected for liver and *longissimus* muscle (**LM**) biopsy as described in the companion manuscript (Harvey et al., 2021). Heifers were observed daily for BRD signs from days 437 to 620 according to Berry et al. (2004).

#### Steer calves

Steer BW was recorded on days 492 and 493, and averaged as final backgrounding BW. Steer ADG during backgrounding was calculated using preconditioning BW and final backgrounding BW. Steers were observed daily for BRD signs from days 437 to 493 according to Berry et al. (2004), and according to the DART system (Zoetis) from day 493 until slaughter based on the management criteria of the feedyard. On day 586, 15 steers from each treatment were randomly selected for liver and LM biopsies (Harvey et al., 2021). At the commercial packing plant, hot carcass weight (**HCW**) was collected upon slaughter. Final finishing BW was estimated based on HCW adjusted to a 63% dressing percentage (Loza et al., 2010). After a 24-hr chill, trained personnel assessed carcass characteristics including backfat thickness at the 12th-rib, marbling, and LM area.

### Laboratorial analyses

All feed samples were analyzed for concentrations of crude protein, acid detergent fiber, neutral detergent fiber, macro-, and trace minerals as described in the companion manuscript (Harvey et al., 2021). Calculations for total digestible nutrients used the equation proposed by Weiss et al. (1992), and calculations for net energy for maintenance and gain used the equations proposed by NRC (2000). Liver and LM samples were immersed in 1 mL of RNA stabilization solution (RNAlater, Ambion, Inc., Austin, TX), placed immediately on ice after collection, and then at −80 °C. Total RNA was extracted, quantity and quality of isolated RNA were assessed, and reverse transcription of extracted RNA and real-time reverse-transcription polymerase chain reaction were completed using the procedures described in the companion manuscript (Harvey et al., 2021), and the gene specific primers (20 pM each) described in Table 3.

**Table 3.** Primer sequences, accession number, and reference for gene transcripts analyzed by real-time reverse transcription polymerase-chain reaction

Target^1^	Primer sequence	Accession#	Source
Liver samples			
CUT			
Forward	GGGTACCTCTGCATTGCTGT	NM_001100381	Han et al. (2009)
Reverse	ATGGCAATGCTCTGTGATGT		
MT			
Forward	ATCCGACCAGTGGATCTGTTTGCC	NM_001040492.2	Gessner et al. (2013)
Reverse	AGACACAGCCCTGGGCACACT		
SOD			
Forward	TGTTGCCATCGTGGATATTG	NM_174615	Gessner et al. (2013)
Reverse	CAGCGTTGCCAGTCTTTGTA		
*Ribosomal protein L12*			
Forward	CACCAGCCGCCTCCACCATG	NM_205797.1	Gessner et al. (2013)
Reverse	CGACTTCCCCACCGGTGCAC		
*Cyclophilin*			
Forward	GGTACTGGTGGCAAGTCCAT	NM_178320.2	Moriel et al. (2014)
Reverse	GCCATCCAACCACTCAGTCT		
*Longissimus* muscle samples			
FABP4			
Forward	AAACTTAGATGAAGGTGCTCTGG	AJ4160220	Li et al. (2018)
Reverse	CATAAACTCTGGTGGCAGTGA		
*Myogenin*			
Forward	GAGAAGCGCAGACTCAAGAAGGTGAATGA	AF09174	Muroya et al. (2002)
Reverse	TCTGTAGGGTCCGCTGGGAGCAGATGATC		
PAX7			
Forward	GGGCTCAGATGTGGAGTCAG	XM_616352.6	Moriel et al. (2014)
Reverse	GCTCCTCTCGGGTGTAGATG		
PPAR-γ			
Forward	GCATTTCCACTCCGCACTAT	AY137204	Li et al. (2018)
Reverse	GGGATACAGGCTCCACTTTG		
*β-Actin*			
Forward	AGCAAGCAGGAGTACGATGAGT	NM_173979	Bong et al. (2012)
Reverse	ATCCAACCGACTGCTGTCA		
*Ribosomal protein L12*			
Forward	CCTCGACCAAGAGCTGAAG	AF479289	Jeong et al. (2012)
Reverse	CCTCCAGACCTCACGTTTGTTC		

^1^CUT, Cu-transporter protein; MT, metallothionein 1A; SOD, superoxide dismutase 1; FAPB4, adipocyte fatty acid-binding protein; PAX7, paired box gene 7; PPAR-γ, peroxisome proliferator-activated receptor-γ.

Blood samples were collected via jugular venipuncture into commercial heparinized blood collection tubes (Vacutainer, 10 mL; Becton Dickinson, Franklin Lakes, NJ). Samples were immediately placed on ice after collection, centrifuged (2,500 × *g* for 30 min; 4 °C) for plasma harvest, and stored at −80 °C on the same day of collection. Samples collected on days 345, 367, 377, and 382 were analyzed for antibodies against bovine herpesvirus-1 (**BHV-1**; BHV-1 Ab ELISA number 99-41459; IDEXX) and bovine viral diarrhea viruses type I and II (**BVDV**; BVDV Ab Elisa number 99-44000; IDEXX Switzerland AG, Liebefeld-Bern, Switzerland) as by Schubach et al. (2020). Samples collected on days 367, 368, 370, 373, 377, 382, and 397 were analyzed for cortisol (radioimmunoassay kit #07221106, MP Biomedicals, Santa Ana, CA; Burdick et al., 2009) and haptoglobin concentrations (Cooke and Arthington, 2013). Samples collected weekly from heifers (days 437 to 620) were analyzed for progesterone concentrations (radioimmunoassay kit #07-170105, MP Biomedicals, Santa Ana, CA; Pohler et al., 2016). Heifers were considered pubertal once plasma progesterone concentrations were ≥1.0 ng/mL followed by a cyclic pattern of plasma progesterone < and ≥ 1.0 ng/mL, suggestive of normal estrous cycles (Schubach et al., 2017). Heifer age and BW at puberty were calculated based on weekly full BW measurements and heifer age at the week of puberty attainment. The intra- and interassay CV were, respectively 4.0% and 4.6% for cortisol, 4.7% and 7.4% for haptoglobin, 6.1% and 5.1% for BHV-1, 1.1% and 4.9% for BVDV, and 6.7% and 11.6% for progesterone.

### Statistical analysis

All data were analyzed with cow as the experimental unit, cow(treatment × parity) as the random variable, using gestation days receiving treatment as an independent covariate. Quantitative data were analyzed using the MIXED procedure of SAS (SAS Inst. Inc., Cary, NC), and binary data were analyzed using the GLIMMIX procedure of SAS (SAS Inst. Inc.). Model statements for responses obtained when heifers and steers were managed together included the effects of treatment, parity, calf sex, day for repeated measures, and all resultant interactions. Model statements for responses specific to heifers or steers included the effects of treatment, parity, day for repeated measures, and all resultant interactions. For all repeated measures, the subject for the repeated statement was cow(treatment × parity) and the covariance structure utilized was autoregressive, which provided the best fit according to the lowest Akaike information criterion. Results are reported as covariately-adjusted least square means, and separated using least square difference. Significance was set at *P* ≤ 0.05, and tendencies were determined if *P* > 0.05 and ≤ 0.10. Results are reported according to main treatment effects if higher-order interactions containing treatments were nonsignificant, or according to highest-order interaction detected.

## Results and Discussion

### Preconditioning period

**Figure 1.**
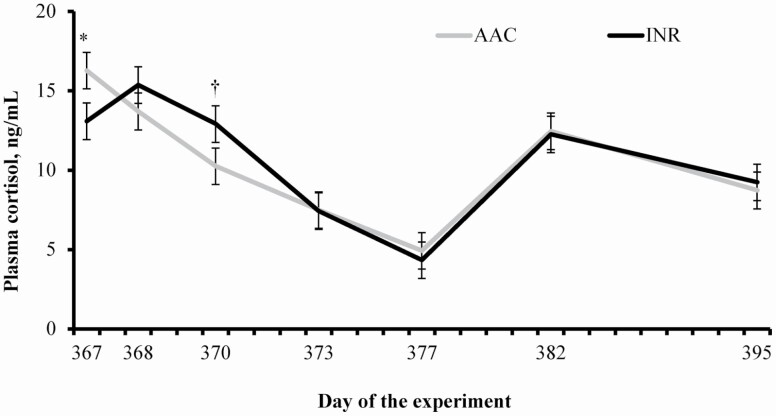
Plasma cortisol concentration of weaned calves (day 367 of the experiment) from beef cows supplemented with sulfate sources (INR; *n* = 95) or organic complexed sources (AAC; *n* = 95) of Co, Cu, Mn, and Zn during gestation. Cows were assigned to the experiment at 117 ± 2 d of gestation (day 0 of the experiment). Calves were weaned on day 367 (88 calves from INR cows and 89 calves from AAC cows) and assigned to a 45-d preconditioning period as a single group until day 412. Plasma was collected from 30 calves randomly selected from each treatment. A treatment × day interaction was detected (*P* = 0.03). Within days: * *P* ≤ 0.05 and † *P* ≤ 0.10.

A treatment × day interaction was detected (*P* = 0.03) for plasma cortisol concentrations, which was greater (*P* = 0.05) in calves from AAC cows at weaning (day 367) but tended to be less (*P* = 0.10) on day 370 compared with calves from INR cows (Figure 1). Mean plasma haptoglobin concentration was greater (*P* = 0.03) in calves from INR cows compared with AAC cohorts (Table 4), while plasma haptoglobin increased in calves from both treatments after weaning (day effect, *P* < 0.01; Table 5). The day effect noted for haptoglobin was expected, based on the acute-phase protein reaction elicited by the weaning process (Schubach et al., 2020). Cortisol has been positively associated with plasma haptoglobin concentrations in cattle (Cooke and Bohnert, 2011; Cooke et al., 2012), although AAC calves had less plasma haptoglobin during preconditioning despite greater plasma cortisol concentrations at weaning compared with INR cohorts. As reported in the companion manuscripts (Harvey et al., 2021), AAC calves had greater mRNA expression of *copper-zinc*-*superoxide dismutase 1* (**SOD**) at weaning; and enzyme that protects cells from oxidative damage by eliminating reactive oxygen species (**ROS**; Tsang et al., 2014). Oxidative stress has also been shown to trigger inflammatory reactions in cattle, including the acute-phase response (Sordillo and Aitken, 2009). Overproduction of ROS can be elicited by environmental factors such as psychological and physiological stressors (Møller et al., 1996), and cortisol has been shown to participate in ROS elimination (Dandona et al., 1999). These results suggest that AAC supplementation to gestating beef cows modulated offspring hepatic metabolism and steroidogenesis required to cope with the stress elicited by weaning, alleviating the resultant acute-phase protein response (Cooke, 2017). Marques et al. (2016) did not report differences in plasma cortisol and haptoglobin between offspring weaned from cows supplemented with sulfate or organic complexed Cu, Co, Zn, and Mn during late-gestation. Perhaps organic complexed trace minerals need to be supplemented longer than the last-trimester of gestation to yield the responses noted herein. Accordingly, Long et al. (2010) reported that adrenal steroidogeneses of the offspring was influenced by maternal nutrition during early gestation.

**Table 4.** Performance and physiological responses during preconditioning in calves from beef cows supplemented with sulfate sources (INR; *n* = 95) or organic complexed sources (AAC; *n* = 95) of Co, Cu, Mn, and Zn during gestation^1^

Item	INR	AAC	SEM	*P-*value
Calf performance				
Treated for BRD symptoms,^2^ %	1.17	0.00	0.810	0.40
Average daily gain,^3^ kg/d	0.563	0.554	0.020	0.76
Final BW, kg	206	201	3	0.23
Plasma variables^4^				
Haptoglobin, mg/dL	0.422	0.326	0.031	0.03
*Bovine viral diarrhea viruses type I and II*	74.2	64.9	5.31	0.22
*Bovine herpesvirus-I*	175	168	6.0	0.40

^1^INR and AAC cows received the same amount of supplemental Co, Cu, Mn, and Zn from sulfate sources or Availa4 (Zinpro Corporation, Eden Prairie, MN). Cows were assigned to the experiment at 117 ± 2 d of gestation (day 0). Calves were weaned on day 367 and assigned to a 45-d preconditioning period as a single group until day 412 (88 calves from INR cows and 89 calves from AAC cows). Calves were vaccinated against respiratory viruses on day 345 (Triangle 5; Boehringer Ingelheim Animal Health USA Inc., Duluth, GA) and day 367 (Titanium 5; Elanco Animal Health, Greenfield, IN).

^2^Calves were observed daily for BRD signs from days 367 to 412 according to the subjective criteria described by Berry et al. (2004), and received 1 mL/10 kg of BW of Baytril 100 (Bayer Animal Health, Shawnee Mission, KS) if diagnosed with BRD.

^3^Calculated based on weaning BW (average of days 367 and 368) and preconditioning BW (average days 411 and 412).

^4^Blood samples were collected on days 345, 367, 368, 370, 373, 377, 382, and 397. Samples collected on days 367, 368, 370, 373, 377, 382, and 397 were analyzed for plasma haptoglobin. Samples collected on days 345, 367, 377, and 382 were analyzed for plasma antibodies against BRD viruses, and results expressed as % sample:positive control ratio as in Colombo et al. (2020).

**Table 5.** Plasma concentrations of haptoglobin (mg/dL), and antibodies against *BVDV type I and II* and BHV in beef calves^1^

Day	BVDV	BHV	Haptoglobin
345	22.8^b^	109^c^	—
367	33.2^b^	151^b^	0.400^c^
368	—	—	0.571^b^
370	—	—	0.774^a^
373	—	—	0.335^cd^
377	—	—	0.204^e^
382	114^a^	216^a^	0.220^de^
397	109^a^	212^a^	0.113^e^
SEM	5.50	8	0.048
*P-*value	<0.01	<0.01	<0.01

^1^Within columns, values with different superscripts differ (*P* ≤ 0.05). Serum antibodies expressed as % sample:positive control ratio as in Colombo et al. (2020). Calves (*n* = 60) received vaccination against respiratory viruses prior to weaning on day 345 (Triangle 5; Boehringer Ingelheim Animal Health USA Inc., Duluth, GA), and at weaning on day 367 (Titanium 5; Elanco Animal Health, Greenfield, IN).

No treatment effects were detected (*P* ≥ 0.22) for plasma concentrations of antibodies against BVDV or BHV-1 (Table 4), which also increased (day effect; *P* < 0.01) in calves from both treatments during preconditioning (Table 5). This day effect denotes that calves effectively acquired humoral immunity against BVDV or BHV-1 upon vaccination (Richeson et al., 2008). Vaccine efficacy is often reduced when administered to stressed animals, given that adrenocortical and acute-phase protein responses impair humoral responses required for immunological memory (Blecha et al., 1984; Munck et al., 1984; Biolatti et al., 2005). Despite differences noted in plasma cortisol and haptoglobin herein, supplementing AAC or INR to gestating beef cows resulted in similar vaccine efficacy in the offspring. Maternal nutrition has been shown to impact offspring humoral response to vaccination against BRD pathogens (Moriel et al., 2016); however, research is still limited and warranted in this area (Cooke, 2019).

Incidence of BRD during preconditioning did not differ (*P* = 0.40) between treatments (Table 4). In fact, BRD incidence was minimal compared to previous research with preconditioning cattle (Marques et al., 2016; Silva et al., 2018), which may have hindered proper assessment of this response. Nonetheless, BRD incidence during on-ranch preconditioning programs is expected to be minimal (Schubach et al., 2020), as calves are not exposed to additional stressors besides the weaning process (Cooke, 2017). No treatment effects were detected (*P* ≥ 0.23) for calf ADG and BW during preconditioning (Table 4). Corroborating these outcomes, Marques et al. (2016) reported similar BRD incidence and ADG during preconditioning in calves from cows supplemented with organic complexed or inorganic Cu, Co, Zn, and Mn during late-gestation. Oxidative stress and acute-phase reaction, however, have been negatively associated with cattle performance responses such as BW gain (Cooke, 2017; Deters and Hansen, 2020). Therefore, treatment effects noted for hepatic mRNA expression of SOD and plasma haptoglobin were not sufficient to improve ADG and BW during preconditioning of calves from AAC cows compared with INR cohorts.

### Heifer responses

**Figure 2.**
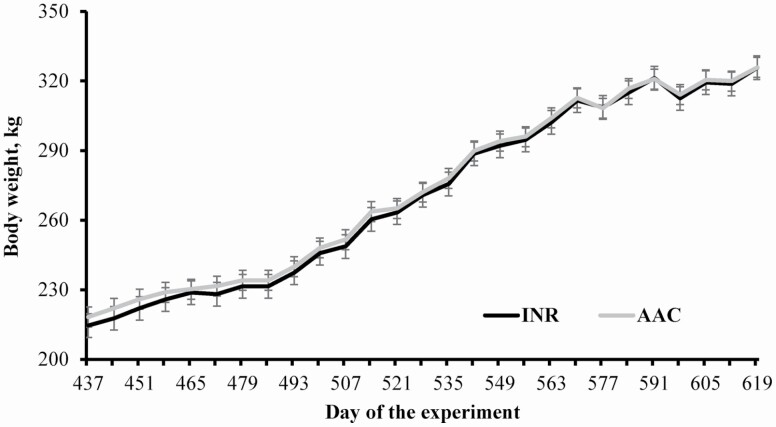
Body weight of replacement heifers from beef cows supplemented with sulfate sources (INR; *n* = 95) or organic complexed sources (AAC; *n* = 95) of Co, Cu, Mn, and Zn during gestation. Cows were assigned to the experiment at 117 ± 2 d of gestation (day 0 of the experiment). Heifers were weaned on day 367 (202 ± 3 d of age), preconditioned for 45 d (days 367 to 412), and managed as a single group on pasture (*Avena sativa* L.) until day 620 (33 heifers from INR cows and 45 heifers from AAC cows). Growth rate of each heifer was modeled by linear regression of BW against sampling days, and each regression coefficient was used as individual response. No treatment differences were noted for BW (*P* ≥ 0.32) or growth rate from days 437 to 619 (0.68 vs. 0.67 kg/d for INR and AAC heifers, respectively; SEM = 0.01).

No treatment differences were detected (*P* ≥ 0.37) for heifer ADG calculated from days 412 to 620 or final BW (Table 6). Similarly, heifer BW and growth rate according to weekly measurements did not differ (*P* ≥ 0.32) between treatments (Figure 2). No incidence of BRD was noted from days 412 to 620, which should be attributed to the preconditioning program and pasture-based system used to develop heifers (Duff and Galyean, 2007). No treatment effects were detected (*P* ≥ 0.48) for mRNA expression of hepatic *Cu-transporter protein* (**CUT**), *metallothionein 1A* (**MT**), and SOD (Table 7), nor *peroxisome proliferator-activated receptor gamma* (**PPAR-γ**) and *adipocyte fatty acid-binding protein* (**FABP4**) in the LM samples collected on day 584 (Table 8). As described in greater extent by the companion manuscript (Harvey et al. 2021), liver CUT, MT, and SOD are associated with Cu and Zn metabolism, whereas PPAR-γ and FABP4 are associated with adipocyte differentiation in the LM. Hence, supplementing AAC or INR to gestating beef cows yielded similar mRNA expression of genes associated with LM adipogenesis and hepatic trace mineral metabolism in the female offspring. In turn, heifers from INR cows had greater mRNA expression of *myogenin* in the LM, and tended to have greater (*P* = 0.09) mRNA expression of *paired box gene 7* (**PAX7**) in the LM compared with ACC cohorts on d 584 (Table 8). *Myogenin* is a regulatory factor that influences postnatal muscle growth through differentiation and fusion of satellite cells with existing fibers (Le Grand and Rudnicki, 2007; Du et al., 2010), while PAX7 is necessary for satellite cell specification and survival (Seale et al., 2000; Li et al., 2011). It is plausible that heifers from INR cows had a greater population of satellite cells undergoing differentiation on day 584. These outcomes, however, were not sufficient to impact heifer growth during their developmental period (days 412 to 620).

**Table 6.** Growth and reproductive responses of replacement heifers from beef cows supplemented with sulfate sources (INR; *n* = 95) or organic complexed sources (AAC; *n* = 95) of Co, Cu, Mn, and Zn during gestation^1^

Item	INR	AAC	SEM	*P-*value
Initial BW,^2^ kg	202	197	5	0.61
Final BW,^2^ kg	332	326	5	0.37
Average daily gain,^2^ kg/d	0.618	0.604	0.011	0.39
Final puberty attainment,^3^ %	83.5	86.4	5.1	0.49
Age at puberty, d	418	399	6	0.04
Body weight at puberty, kg	319	310	6	0.24

^1^INR and AAC cows received the same amount of supplemental Co, Cu, Mn, and Zn from sulfate sources or Availa4 (Zinpro Corporation, Eden Prairie, MN). Cows were assigned to the experiment at 117 ± 2 d of gestation (day 0). Heifers were weaned on day 367 (202 ± 3 d of age), preconditioned for 45 d (days 367 to 412), and managed as a single group on pasture (*Avena sativa* L.) pasture until day 620 (33 heifers from INR cows and 45 heifers from AAC cows)

^2^Heifer initial and final BW were calculated, respectively, according to the average of BW recorded at the end of preconditioning (days 411 and 412) and average of BW recorded on days 619 and 620. Average daily gain was calculated using initial and final BW.

^3^ Evaluated according to plasma progesterone concentrations in samples collected weekly from days 437 to 619 (Schubach et al., 2017).

**Table 7.** Expression of liver genes in calves from beef cows supplemented with sulfate sources (INR; *n* = 95) or organic complexed sources (AAC; *n* = 95) of Co, Cu, Mn, and Zn during gestation^1,2^

Item^3^	INR	AAC	SEM	*P-*value
CUT				
Heifer	2.01	1.89	0.12	0.48
Steer	1.91	2.01	0.11	0.50
MT				
Heifer	126.4	161.2	63.0	0.71
Steer	5.35	3.08	0.63	0.02
SOD				
Heifer	2.17	2.15	0.16	0.95
Steer	1.92	2.01	0.11	0.58

^1^INR and AAC cows received the same amount of supplemental Co, Cu, Mn, and Zn from sulfate sources or Availa 4 (Zinpro Corporation, Eden Prairie, MN). Cows were assigned to the experiment at 117 ± 2.2 d of gestation (day 0). Calves were weaned on day 367 of the experiment at 200 ± 2 d of age and preconditioned as a single group until day 412. Heifers were managed as a single group on pasture (*Avena sativa* L.) until day 620 (33 heifers from INR cows and 45 heifers from AAC cows). Steers were also managed as a single group on pasture (*Avena sativa* L.) until day 493, when they were transported to a commercial feedlot (Graham Land and Cattle Company; Gonzales, TX) where they remained as a single group until slaughter (day 724; 50 steers from INR cows and 44 steers from AAC cows).

^2^Liver samples were collected from 30 animals randomly selected per treatment (Arthington and Corah, 1995) on day 584 (15 heifers/treatment) and from steers (15 steers/treatment) on day 586. Values are expressed as relative fold change compared within threshold cycle of reference genes analyzed within the same sample (Ocón-Grove et al., 2008).

^3^CUT, Cu-transporter protein; MT, metallothionein 1A; SOD, superoxide dismutase 1.

**Table 8.** Expression of LM genes in calves from beef cows supplemented with sulfate sources (INR; *n* = 95) or organic complexed sources (AAC; *n* = 95) of Co, Cu, Mn, and Zn during gestation^1,2^

Item^3^	INR	AAC	SEM	*P-*value
FABP4				
Heifer	4.68	5.10	1.5	0.85
Steer	7.56	7.12	1.3	0.82
*Myogenin*				
Heifer	4.59	2.87	0.58	0.05
Steer	2.98	2.56	0.28	0.30
PAX7				
Heifer	1.91	1.70	0.08	0.09
Steer	1.67	1.56	0.11	0.51
PPAR-γ				
Heifer	1.62	1.53	0.21	0.77
Steer	2.22	2.52	0.30	0.49

^1^INR and AAC cows received the same amount of supplemental Co, Cu, Mn, and Zn from sulfate sources or Availa 4 (Zinpro Corporation, Eden Prairie, MN). Cows were assigned to the experiment at 117 ± 2.2 d of gestation (day 0). Calves were weaned on day 367 of the experiment at 200 ± 2 d of age and preconditioned as a single group until day 412. Heifers were managed as a single group on pasture (*Avena sativa* L.) until day 620 (33 heifers from INR cows and 45 heifers from AAC cows). Steers were also managed as a single group on pasture (*Avena sativa* L.) until day 493, when they were transported to a commercial feedlot (Graham Land and Cattle Company; Gonzales, TX) where they remained as a single group until slaughter (day 724; 50 steers from INR cows and 44 steers from AAC cows).

^2^Muscle samples were collected from 30 animals randomly selected per treatment (Schubach et al., 2019) on day 584 (15 heifers/treatment) and from steers (15 steers/treatment) on day 586. Values are expressed as relative fold change compared within threshold cycle of reference genes analyzed within the same sample (Ocón-Grove et al., 2008).

^3^FABP4, adipocyte fatty acid binding protein; PAX7, paired box gene 7; PPAR-γ, peroxisome proliferator-activated receptor-γ.

**Figure 3.**
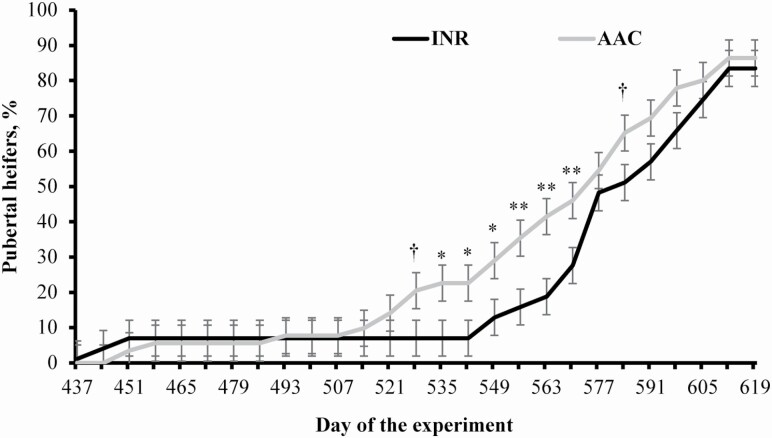
Puberty attainment of replacement heifers from beef cows supplemented with sulfate sources (INR; *n* = 95) or organic complexed sources (AAC; *n* = 95) of Co, Cu, Mn, and Zn during gestation. Cows were assigned to the experiment at 117 ± 2 d of gestation (day 0 of the experiment). Heifers were weaned on day 367 (202 ± 3 d of age), preconditioned for 45 d (days 367 to 412), and managed as a single group on pasture (*Avena sativa* L.) until day 620 (33 heifers from INR cows and 45 heifers from AAC cows). Puberty was evaluated according to plasma progesterone concentrations in samples collected weekly from days 437 to 619 (Schubach et al., 2017). A treatment × day interaction was detected (*P* < 0.01). Within days: † 0.05 ≤ *P* ≤ 0.10; * *P* ≤ 0.05; ** *P* < 0.01.

A treatment × day interaction was detected (*P* < 0.01) for puberty attainment, as heifers from AAC cows reached pubertal earlier in the experiment compared with INR cohorts (Figure 3). Final puberty attainment and BW at puberty did not differ (*P* ≥ 0.24) between treatments, whereas heifers from AAC cows were younger (*P* = 0.04) at puberty compared with INR cohorts (Table 6). Onset of puberty is highly modulated by body composition and development (Schillo et al., 1992), while attainment and age at puberty differed between heifers from AAC and INR cows despite similar BW gain. Fat accretion also impacts puberty in heifers (Yelich et al., 1995), and mRNA expression of *adipogenic* genes in the LM did not differ between treatments on day 584. The intramuscular region, however, is the last depot for adipogenesis in growing cattle (Oliveira et al., 2011), and the puberty process is likely more influenced by subcutaneous fat accretion (Lammoglia et al., 2000; Garcia et al., 2002; Roberts et al., 2017). In turn, myogenic factors are downregulated as cattle mature and muscle fibers are fully developed (Picard et al., 2002; Du et al., 2010); hence, treatment differences noted for mRNA expression of *myogenin* and PAX7 in the LM (day 584) may be indicative of hastened physiological maturation in heifers from AAC cows (Gonzalez et al., 2020).

Previous research demonstrated that supplementing organic complexed trace minerals improved reproductive performance in dairy cattle, such as decreased interval to first estrus and increased pregnancy rates (Uchida et al., 2001; Griffiths et al., 2007). In beef cows, supplementing organic complexed Co, Cu, Mn, and Zn resulted in greater number of culturable oocytes and transferable embryos from *in vitro* fertilization compared with cohorts receiving sulfate sources (Dantas et al., 2019). These latter results may help explaining the hastened puberty attainment of heifers from AAC cows in this experiment. Primordial germ cells present in the developing ovary actively utilize machinery and enzymes against ROS to maintain cell integrity (Hayashi et al., 2017), and trace mineral supplementation increased antioxidant enzyme activity and decreased ROS production during germ cell development (Özkaya and Nazıroğlu, 2010; Shi et al., 2010). Trace mineral deficiency, particularly Zn, has also been associated with epigenetic defects in oocytes and impaired ovarian development during the fetal period (Hurley and Keen, 1988). Therefore, supplementing AAC to gestating cows may have favored ovarian development of the heifer offspring, protecting ovarian cells and follicles from endogenous ROS compared with INR-supplemented cows. Although research is required to validate this rationale, results from this experiment are novel and suggestive of programming effects on heifer reproductive development from supplementing AAC to their dams during gestation.

### Steer responses

No treatment effects were noted (*P=* 0.74) for ADG (0.960 and 0.946 kg/d for steers from INR and AAC cows, respectively; SEM = 0.027) during the backgrounding period (days 412 to 493), nor for BW (*P* = 0.21) at the time of shipping to the feedyard (Table 9). No treatment effects were also detected (*P* ≥ 0.24) for steer ADG and final BW in the feedyard, as well as carcass traits upon slaughter (Table 9). Incidence of BRD did not differ (*P* = 0.56) between steers from AAC and INR cows in the feedyard (Table 9), and BRD was not was detected during the backgrounding period. Hence, supplementing AAC to gestating beef cows did not improve postnatal growth and carcass composition of the male offspring compared with INR, corroborating the BW gain results reported for the heifer offspring. Marques et al. (2016) also reported that feedlot ADG did not differ in heifer and steers from cows supplemented with INR or AAC during late gestation, although incidence of BRD was less in calves from AAC cows. Those authors shipped all offspring to a commercial feedyard after a 45-d preconditioning, and reported elevated BRD incidence during the 110-d growing phase despite the preconditioning program. In this experiment, steers were backgrounded on pasture for 81 d after the 45-d preconditioning period, which likely resulted in the low BRD incidence across treatments (Duff and Galyean, 2007; Table 9) and hindered the assessment of this response in the feedyard.

**Table 9.** Feedlot performance of steers from beef cows supplemented with sulfate sources (INR; *n* = 95) or organic complexed sources (AAC; *n* = 95) of Co, Cu, Mn, and Zn during gestation^1^

Item	INR	AAC	SEM	*P-*value
Feedlot performance				
Shipping BW (day 493), kg	287	279	5	0.21
Final BW (day 724),^2^ kg	590	582	8	0.49
Average daily gain, kg/d	1.29	1.29	0.03	0.98
Treated for BRD symptoms,^3^ %	2.2	4.3	2.5	0.56
Carcass characteristics^4^				
Hot carcass weight, kg	372	367	5	0.49
Backfat, cm	1.63	1.68	0.08	0.72
*Longissimus* muscle area, cm	80.2	80.8	1.0	0.70
Marbling score	407	402	9	0.70
Yield grade	3.74	3.71	0.11	0.85
Carcass graded choice or greater, %	49.8	37.5	7.3	0.24

^1^INR and AAC cows received the same amount of supplemental Co, Cu, Mn, and Zn from sulfate sources or Availa4 (Zinpro Corporation, Eden Prairie, MN). Cows were assigned to the experiment at 117 ± 2 d of gestation (day 0). Steers were weaned on day 367 (197 ± 3 d of age), preconditioned for 45 d (days 367 to 412), managed as a single group on pasture (*Avena sativa* L.) until day 493, and transported to a commercial feedlot (Graham Land and Cattle Company; Gonzales, TX) where they remained as a single group until slaughter (day 724; 50 steers from INR cows and 44 steers from AAC cows).

^2^Calculated based on HCW (assuming 63% dressing; Loza et al., 2010).

^3^Calves were classified as positive for BRD symptoms according to the DART system (Zoetis Inc., Florham Park, NJ) and received medication according to feedlot management criteria.

^4^Back fat thickness measured at the 12th rib. Marbling score: 400, Small^00^ and 500, Modest^00^; yield grade calculated as reported by Lawrence et al. (2010).

Hepatic mRNA expression of MT was greater (*P* = 0.02) in steers from AAC cows compared with INR cohorts on day 586, while no treatment differences were detected (*P* ≥ 0.50) for mRNA expression of CUT and SOD (Table 7). As described in the companion manuscript (Harvey et al., 2021), these genes are associated with Cu and Zn metabolism in the liver, and their hepatic mRNA expression positively associated with hepatic Cu and Zn concentrations (Bauerly et al., 2005; Hansen et al., 2009; Wang et al., 2012). The greater mRNA expression of MT in steers from INR cows could be associated with increased feed intake and consequent accumulation of trace minerals in the liver. Contrariwise, feedlot ADG and mRNA expression of hepatic SOD and CUT did not differ between treatments, whereas upregulation of hepatic MT did not translate into improved performance of INR steers. Expression of FABP4, *myogenin*, PAX7, and PPAR-γ mRNA also did not differ (*P* ≥ 0.30) between treatments (Table 8). These outcomes suggest similar muscle development and adipogenesis in steers from AAC and INR on d 586, and are supported by equivalent phenotypic responses in carcass merit traits between treatments (Table 9). Collectively, results from the male offspring indicate that supplementing gestating beef cows with AAC instead of INR did not promote programing effects on performance and physiological responses of steers reared as feeder cattle and finished in a commercial feedyard.

### Overall conclusions

Supplementing beef cows with organic-complexed sources of Co, Cu, Mn, and Zn during gestation alleviated the acute-phase response in the offspring after weaning, which did not translate into improved calf performance and humoral response to vaccination against BRD pathogens during a 45-d preconditioning period. The steer progeny from cows receiving organic-complexed and sulfate sources had similar performance responses until slaughter, including feedlot ADG and carcass merit traits. In turn, the heifer progeny from cows receiving organic-complexed trace minerals had hastened puberty attainment compared with cohorts from cows that received sulfate sources. These outcomes complement the findings reported by the companion manuscript (Harvey et al., 2021), and suggest that supplementing an organic-complexed source of Co, Cu, Zn, and Mn to gestating beef cows enhances reproductive development of their female offspring raised as replacement heifers.

## Abbreviations

ADG: average daily gain
BHV-1: bovine herpesvirus-1
BRD: bovine respiratory disease
BVDV: bovine viral diarrhea viruses type I and II
BW: body weight
LM: *longissimus* muscle
PCR: polymerase chain reaction
ROS: reactive oxygen species
